# Psychiatric nurses’ experience of aggression amongst colleagues

**DOI:** 10.4102/hsag.v23i0.1086

**Published:** 2018-06-21

**Authors:** Marisa Roets, Marie Poggenpoel, Chris Myburgh

**Affiliations:** 1 Department of Nursing Science, Faculty of Health Sciences, University of Johannesburg, South Africa; 2 Department of Educational Psychology, University of Johannesburg, South Africa

## Abstract

Psychiatric nurses run a high risk of being exposed to aggression. They experience aggression from clients as well as fellow colleagues. Aggression in the work environment has an overt negative psychological effect on the nurse. The purpose of this research was to explore and describe how psychiatric nurses experienced aggression amongst colleagues in the work environment. The study used a qualitative, exploratory and descriptive research design. Eight psychiatric nurses exposed to aggression by their colleagues in an academic psychiatric hospital in Johannesburg were purposively sampled to participate in this study. Data were collected by means of in-depth phenomenological interviews, observations and field notes until data saturation was achieved. The following question was asked: ‘What is your experience of aggression amongst colleagues in the work environment?’. The findings indicated that the psychiatric nurses experienced aggression in a passive but harmful manner. The nurses experienced a suspicious and distrustful team environment. Limited support was experienced when colleagues and management did not acknowledge aggression and the nurses applied various coping and defence mechanisms when emotional stress and aggression were experienced. The aggression psychiatric nurses experienced had an effect on their experience on self, team work and providing services to patients.

## Introduction

Mental health professionals are mostly dedicated carers devoting themselves to people who are suffering from mental illness. The fact that dedicated staff end up possibly creating and supporting institutions and services that may cause harm, is troubling. It is likely that because mental health professionals are some of the most caring people, they become the most affected by being with those who suffer. The mental health professionals do so for long hours and many years. They end up suffering from burnout (Hinshelwood 2016).

Research by Menzies (1959, Republished in 1988) on nurses in general hospitals with physically ill patients, indicated that these nurses were in the presence of patients who were in pain, dying, frightened and scarred by operations. The nursing practice developed as such that the nurses avoided close relationships with patients and to make decisions. They practiced functional nursing. In such practice, nurses related to only a part of each patient. Minor decisions were passed on to be taken at the top of the nursing hierarchy. This also happened in psychiatric hospitals. In psychiatric wards, nurses also avoided personal contact with patients (Donati 1989). The medical model the nurses adhered to, focused on medical diagnosis and led to a process of emotional distancing which is not recognised, but becomes standard practice. It assisted the mental health professionals to cope with the impact of mental illness. It did not assist the patients who suffered from mental illness. It rather increased the patients’ suffering. On the contrary, the suffering of mental health professionals were not recognised by themselves nor was it recognised by the management who were suppose to provide support.

Bilgin (2009) studied the interpersonal skills of nurses and found that nurses are more likely to feel that their rights are ignored, while the rights of patients are maintained and protected. According to Lin, Probst and Hsu (2010), research had identified nursing as a high-stress profession. Nurses cope daily with extreme physical and psychological demands inherent in providing care to acute and chronic populations. The demands of caring for others can be extremely stressful on the psychiatric nurse (Van Rhyn & Gostsana 2004).

According to Van Rhyn and Gostsana (2004), most studies on stress, experienced in a psychiatric setting, focused on staffing levels: overworking and administrative duties. In addition, they face unique challenges in their day-to-day work that reflect in their interaction with a particular patient group. Working with shortages of staff in an inadequate physical working environment and under hierarchical pressure with regard to colleagues, co-workers or medical staff as well as being victims of interpersonal violence, are all indicators of a stressful work environment (Bilgin 2009).

Various studies have been done on aggression in the work environment. Yildirim (2009) states that nurses are at a high risk of being exposed to violence and aggression in the work environment. Both Yildirim (2009) and Bimenyimana et al. (2009) indicated that violence and aggression ultimately had an overt negative psychological effect on the nurses.

## Problem statement

Psychiatric nurses daily interact with other people in their working environment. Psychiatric nurses experience violence and aggression from not only their colleagues, but also from their patients which have negative psychological effects (Bimenyimana et al. 2009; Yildirim 2009). Violence and aggression in psychiatric hospitals have been widely researched internationally and nationally (Bilgin 2009; Bimenyimana et al. 2009).

The importance and effect of violence and aggression in day-to-day living should not be underestimated. It is assumed that it could have severely harmful psychological consequences in the long term and that it can thus affect the psychiatric nurses’ self-esteem, social status and happiness. Bilgin (2009) explains the importance of nursing staff’s roles and attitudes in the interaction with the psychiatric environment which should ultimately strive to create a therapeutic milieu. This implies that the psychiatric nurse’s interpersonal skills play an extremely important role in the creation of such an ideal milieu in the work environment.

The researcher worked as a psychiatric professional nurse in an academic psychiatric hospital in Johannesburg. While working in a psychiatric unit, the researcher observed that, at times, colleagues displayed behaviour such as constant late coming, refusal and/or delaying of participation in tasks and, at times, ignoring one another.

The following research question guided this research: What is the lived experience of the psychiatric nurse of aggression amongst colleagues in the work environment?

## Research purpose

The overall purpose of the study was to explore and describe the lived experience of psychiatric nurses of aggression amongst colleagues in the work environment.

## Definitions of concepts

### Aggression

Aggression refers to a heterogeneous construct with substantial semantic overlap with terms for many forms of behaviour with the intent to harm others (Ramirez 2011). Kaplan and Sadock (2007) are of the opinion that many behaviours are aggressive, even though they do not involve direct physical injury. Examples of this behaviour are verbal aggression, coercion, intimidation and social ostracism of others.

### Interpersonal aggression

Interpersonal aggression is behaviour that goes against the interests of other individuals in the workplace such as interfering with the work of others, getting into arguments, treating others with disrespect and gossiping about others (Lyons 2017).

### Active aggression

Individuals who use active aggression are obvious in the expressions of their emotion. They intimidate and coerce their victims in a show of clear and observable physical aggression. They may display their aggression verbally or non-verbally by glaring at their victims, rolling their eyes at them, shaking their head or tutting when their victims speak, stepping up to their victims with a contemptuous expression on their face and speaking to their victims in an openly irate and rude way (Oade 2015).

### Passive aggression

Persons who use passive aggression may feel just as aggressive as those with active aggression. Passive aggression is expressed in a more subtle and indirect way such as making insulting comments with a smile, opposing the viewpoint of the victims in a understated and reasonable tone, using their influence to ensure that the proposals put forward by the victims are rejected or become subject of a heated debate and undermining the victims’ reputation behind their back through slander (Oade 2015).

### Violence

Violence is behaviour that inflicts harm, damage or injury (English Oxford Living Dictionaries 2018). Bimenyimana et al. (2009) states that violence means any act, word or attitudes, such as an intimidating facial expression, that create fear or negative feelings, leading to or resulting in physical, psychological and social unwanted results.

### Bullying

Workplace bullying constitutes repeated offensive behaviour through vindictive, cruel, malicious or humiliating attempts to undermine an individual or group of employees (Chappell & Di Martino 2006). The victims of bullying are subjected to being terrorised, annoyed, secluded, belittled, deprived of sources, isolated and prevented from claiming rights (Yildirim 2009).

### Burnout

Burnout refers to exhaustion of physical or emotional strength, usually as a result of prolonged stress or frustration (Merriam-Webster Online Dictionary 2017).

## Research design and method

### Research design

The research design of the study was qualitative, exploratory, descriptive and contextual in nature (Streubert & Carpenter, 2011). The researcher sought to inquire into and identify the essence of human experience about a phenomenon as described by a participant (Creswell 2013). The intention for exploring this phenomenon was to capture the psychiatric nurses’ lived experiences of aggression amongst colleagues.

### The setting

The population for this study consisted of the psychiatric nursing staff registered with the South African Nursing Council and was employed in an academic psychiatric hospital in Johannesburg. The researcher utilised purposive sampling. Through purposive sampling information, information rich participants were selected (Sullivan 2009). The criteria for sampling were as follow: participants were professional nurses who were working in an academic psychiatric hospital where the research was conducted, and who had been working there uninterruptedly for a minimum period of 24 months. The psychiatric nurses needed experience in an academic psychiatric hospital in order to give a valid account of their experiences and to be familiar with the environment in which they work. They had to be able to speak English or Afrikaans.

Data saturation occurred (Streubert & Carpenter 2011) after a total of eight registered psychiatric nurses were interviewed. The sample consisted of two males and six females, aged from 24 to 59 years. One of the interviews was an auto-ethno-graphical interview during which the researcher shared her own experience of aggression by colleagues with an interviewer.

## Data collection

Data were collected by means of in-depth phenomenological interviews, observation and field notes (Burns, Grove & Gray 2013; Giorgi 1985). The researcher’s intention was to gain knowledge about the participants’ lived experiences with aggression amongst colleagues. The central question that was asked in the in-depth phenomenological interviews was:

What is your experience of aggression amongst colleagues in the work environment? (Delport 2013:32)

All interviews were recorded and transcribed. Field notes, based on observations during the interviews, were taken. The researcher used communication skills such as probing, clarifying, reflecting on the context, bracketing, minimal response and summarising (Burns et al. 2013).

## Data analysis

The transcribed interviews and field notes were analysed by means of thematic coding (Holloway & Wheeler 2010). The data were coded and linked into themes. The focus in the data analysis was on psychiatric nurses’ lived experience of aggression amongst colleagues. Supporting direct quotations from participants were included in the description of the themes. An experienced independent coder analysed the data separately from the researcher. A consensus discussion was held between the independent coder and the researcher about the findings.

## Trustworthiness

Measures to ensure trustworthiness were applied to this research such as credibility, transferability, dependability and confirmability (Lincoln & Guba 1985). Trustworthiness refers to the acquirement of knowledge and understanding of the true nature, essence, meaning, attributes and characteristics of the phenomenon (Leiniger 1985). Strategies of credibility were ensured by the researcher engaging with participants to the point of data saturation. The data collected from the participants were checked with the participants during and after collection (Krefting 1991). The researcher worked as a professional psychiatric nurse in the same hospital for several years. During data collection, field notes and observations were utilised. Strategies of transferability were ensured by giving a description of the demographic information of the participants as well as a dense description of data, supported by direct quotations from participants (Krefting 1991). Dependability was achieved by means of a dense description of the research methodology used in this research. All interview materials, transcripts, documentation, findings, interpretations and recommendations were kept available and accessible to the supervisors and any other researcher for the purpose of conducting a possible audit trail (Krefting 1991). An audit trail of the verbatim descriptions, themes and categories ensured confirmability.

## Ethical considerations

The study involved human participants and their rights were protected. This was ensured by adhering to the four ethical principles, namely autonomy, non-maleficence, beneficence and justice. Each principle is binding, unless it clashes with an equal or stronger obligation (Dhai & McQuoid-Mason 2011). All participants were given a fair opportunity to choose to participate and were given a right to self-determination. The researcher initially informed the participants about the research study, enabling them to determine whether they want to participate in the study or not. The goal of the research study was explained. The participants’ informed consent was obtained (Burns et al. 2013). The participants’ names were not mentioned during or after the interviews or during transcription and coding. All information received, was treated professionally and with respect. All data collected were kept confidential; this assured the participants of their privacy. Data were collected by means of in-depth phenomenological interviews and naive sketches. Consent was obtained for the use of an audio-recorder. All participants had the choice to opt out of the interview at any stage or refuse to answer specific questions. Recordings were kept under lock and key, with only the researcher and supervisors having access to it. Recordings were destroyed two years after publication of the research. Participants were not identified in the research report. No compensation was paid to any of the participants for participating in the study (Burns et al. 2013). No harm occurred during the course of the research.

This research study was approved by the Faculty of Health Sciences, Research Ethics Committee (AEC 28/02-2011, M110611).

## Findings

The following themes emerged from the study:

The psychiatric nurses experienced aggression as subtle, passive and harmful, manifesting in verbal and non-verbal ways. The psychiatric nurses experienced their team environment as doubtful, suspicious and distrustful which led to questioning themselves, their colleagues and their career as psychiatric registered nurses working in a psychiatric hospital. The psychiatric nurses experienced management as giving limited support which appeared to contribute to the distress and demoralisation of the nurses. The psychiatric nurses experienced management as not acknowledging the aggression they experience. The psychiatric nurses used various coping and defence mechanisms when emotional stress and aggression were experienced (Delport 2013:36-55). The themes will now be discussed in greater detail.

### Psychiatric nurses experienced aggression as subtle, passive and very harmful in verbal and non-verbal ways

The psychiatric nurses explained in the interviews that their experience of aggression affected them deeply and negatively, especially amongst colleagues in the work environment. Psychiatric nurses, as participants, experienced aggression in different forms. The participants reported that aggression manifested in verbal and non-verbal forms at times. As the data emerged, it appeared as if the participants experienced aggression amongst colleagues in a subtle, passive and very harmful manner. A participant reported that:

‘I have witnessed a lot of passive aggression and underlying hostility amongst colleagues.’ (Participant # 2)

During the interviews, the psychiatric nurses explained that their experiences of aggression manifested in a verbal form such as when colleagues gossip, make snide remarks or make inappropriate comments in the work environment. The psychiatric nurses explained that this was a harmful and disrespecting experience for them. One participant explained that:

‘It’s like talking behind each other’s back and it’s like being negative towards them …’. (Participant # 5)

Psychiatric nurses experienced language abuse and cultural differences as aggression. These differences often resulted in a harmful feeling of isolation and being ignored. This also led to participants being suspicious of their colleagues which, in turn, affected the team negatively. One participant said

‘Who I work with, their first language is not my first language, they tend to converse in their first language.’ (Participant # 6)

Another participant added that:

‘They do it to kind of provoke me, they sometimes do it to rat against me.’ (Participant # 1)

In addition, psychiatric nurses described that their experiences of aggression manifested in a non-verbal manner. Participants described the non-verbal behaviour as being passive aggressive. This was highlighted by a participant who said:

‘I felt a lot of passive aggression in a new ward I was allocated to. Staff members would sit with their back to me, and blocking me out of conversations.’ (Participant # 3)

The participants described that their colleagues ignored and judged this aggressive behaviour in the work environment, resulting in being treated unprofessionally. This led to feelings of apprehension about working in a team which led to participants choosing rather to work in isolation. Participants verbalised that body language and staring looks could communicate aggression to them. The following statement explained what the participants experienced:

‘… cold shoulder, not speaking to you, passive aggressive looks … look at you in a way, but people really do look’. (Participant # 8)

### Psychiatric nurses experienced a doubtful, suspicious and distrustful team environment

The psychiatric nurses experienced their team environment as doubtful, suspicious and distrustful when they were confronted with aggression. The environment was experienced as so toxic that it created a ‘paranoid’ team environment. This toxic and paranoid environment resulted in the psychiatric nurses questioning themselves. This consequently affected the psychiatric nurses treating themselves without respect and in a poor manner. This had the effect that the psychiatric nurses did not enjoy their work and it influenced service delivery to clients. The psychiatric nurses verbalised how ineffectual they felt in this seemingly paranoid environment. It resulted in the psychiatric nurse having a poor self-esteem and decreased confidence which, in turn, affected the quality of their work. Participant explained that:

‘… you burn out, you feel unwell you feel not cared for …’ (Participant # 7)

‘… it was hard to function effectively …’ (Participant # 4)

‘… lowered self-esteem … ‘ (Participant # 3)

Because of the aggression, the psychiatric nurses experienced their team environment as doubtful, suspicious and distrustful. They therefore questioned their colleagues’ behaviour towards them. The consequences of questioning their colleagues’ behaviour were that they did not trust each other and did not care for each other in the work environment. The feelings of not being cared for resulted in psychiatric nurses questioning themselves and thus leaving them feeling insecure which led to easy intimidation by each other. This had an effect on team work and contributed to the lack of quality of care in the work environment. The psychiatric nurses explained that:

‘… it is more of a neglect of each other and colleagues …’. (Participant # 1)

‘It does not feel like a team …’. (Participant # 6)

‘… duty room became “hot”’. (Participant # 2)

With a lack of trust in the interaction between psychiatric nurses in the nursing team, the nurses began questioning their career on a daily basis. Their experience of aggression made them feel frustrated and challenged. Anger and resentment contributed to the psychiatric nurses loosing passion for their daily duties: not wanting to go to work or finding excuses to be absent. Because of the lacking of motivation, they became demoralised and sought other more fulfilling career opportunities. The following statement from participants supported this:

‘… you harbour resentment, you lose your passion, you resent coming to work’. (Participant # 5)

‘… seeking greener pastures’. (Participant # 8)

### Psychiatric nurses experienced limited support when aggression was not acknowledged by colleagues and management

Psychiatric nurses experienced limited support in the work environment, apparently because of aggression being present. The most evident experiences of aggression were associated with limited or no support, by not acknowledging each other, avoiding each other’s feelings and difficult circumstances. The psychiatric nurses verbalised that they did not feel supported by their colleagues and/or the management that they worked with every day. In this research study, the psychiatric nurses interviewed, saw management as their colleagues in the work environment. The following quotations supported this:

‘… not a lot of support …’. (Participant # 1)

‘… We don’t support each other, and if you like to support there is something wrong with you.’ (Participant # 3)

The psychiatric nurses reported that their experience of no support from management contributed to feelings of being judged in the work environment. According to the psychiatric nurses, aggression manifested itself in management’s continuous reprimands and constant threats of disciplinary actions and performance appraisals. The psychiatric nurses constantly expressed that their experience of management, not recognising their work, made them feel despondent towards the work environment. The following comments supported this:

‘A lot of emphasis on discipline.’ (Participant # 5)

‘… management is neglectful of us, they are not interested, they are ignorant’. (Participant # 6)

Further contributing to distress, the psychiatric nurses experienced pressure, a lack of resources and unfair assignment of duties. The participants related the lack of resources in the work environment to a shortage of staff, a lack of equipment and a lack of in-service training. Psychiatric nursing staff noted that one of the factors contributing to aggression in the working environment was the unfair assignment of duties or rotation of nursing staff to other wards without consulting or informing them prior to rotation. A large number of nursing staff coped with conflict by finding a new place of employment which, in turn, resulted in an increase in staff turnover.

The participants verbalised that:

‘… shortage of staff …’. (Participant # 7)

‘… rotate the duties …’. (Participant # 4)

‘… incompetent toward some procedures’. (Participant # 2)

and

‘… not included in decisions is aggressive …’. (Participant # 5)

### Psychiatric nurses experienced using coping and defence mechanisms when emotional stress and aggression were experienced

Psychiatric nurses experienced emotional stress as a result of aggression in the work environment and used different coping mechanisms to survive in this environment. When the psychiatric nurses experienced emotional stress, they felt overwhelmed and experienced burnt out. The emotional stress in the work environment led to various factors affecting the individual. One of the emotional stressors of the psychiatric nurse was frustration which could lead to aggression in the work environment. The psychiatric nurses explained that they experienced:

‘… enough emotional damage …’. (Participant # 2)

‘… I am stressed, burnout … I am not a machine …’. (Participant # 6)

and

‘… needs of staff not being met … emotional needs …’. (Participant # 1)

The psychiatric nurses experienced disappointment, which led to mistrust and effectively negated teamwork in the work environment, when aggression was present in the work environment. Aggression ultimately led to resentment in the work environment. When the nurses experienced constant negative stress and overwhelming emotions, the team started feeling demoralised and tension built up, allowing aggression to evolve. The following statements were made by the psychiatric nurses:

‘… don’t seem to care, everyone for himself …’. (Participant # 8)

‘… can’t see each other’s struggles …’. (Participant # 5)

‘… no one has time …’. (Participant # 4)

‘constant tension, resentment and aggression …’. (Participant # 7)

When the psychiatric nurses experienced overwhelming emotional stress and aggression in the work environment, they found a means of coping with their feelings. The psychiatric nurses managed and coped with aggression by means of defence mechanisms. The most general defence mechanism was suppression: not wanting to manage conflict or aggression in the work environment. The psychiatric nurses projected anger and aggression when they experienced aggression in the work environment. Withdrawal, isolation, suppression, projection and passive aggressiveness were defence mechanisms found to be used by various psychiatric nurses when they were overwhelmed and experienced emotional stress and aggression. Participants said the following about avoiding aggression:

‘I don’t address it …’. (Participant # 5)

‘… displace the anger …’. (Participant # 3)

‘… ek bly meeste van die tyd uit hulle pad …’. [‘*I stay away from them most of the time*’]. (Participant # 7)

Psychiatric nurses experienced that aggression in the work environment tended to be overwhelming and coped by means of passive aggressive behaviour. The passive aggressive behaviour noted was that psychiatric nurses tended to be absent from work - with or without excuse - resulting in high absenteeism. They were unproductive at work, not delegating duties or not appointing themselves for any duties. At times, the professional nurses took revenge on each other with the intent to punish or cause harm. The participants explained the following:

‘… don’t want to come to work …’. (Participant # 2)

‘… you become unproductive …’. (Participant # 4)

‘… not performing delegating duties …’. (Participant # 8)

‘… you do things with an attitude …’. (Participant # 6)

‘… you make statements … a way of getting them …’. (Participant #1)

## Discussion of findings

The psychiatric nurses experienced aggression as subtle, passive and harmful in a verbal and non-verbal manner. The psychiatric nurses reported that different types of aggression were present in their work environment, namely verbal and non-verbal aggression. Verbal aggression was visible in the forms of gossiping, backbiting, snide remarks made to each other, the abuse of language barriers and cultural differences. The nurses denied direct or physical aggression in their work environment and reported that only in selective cases they had the urge to react at a physical level. All the psychiatric nurses emphasised the experience of non-verbal aggression in the work environment. Non-verbal aggression manifested in the psychiatric nurses’ reports of feeling ignored and judged by their colleagues. They experienced glaring looks and having backs turned on them as in aggressive body language.

Various studies done internationally and nationally focused on the violence and aggression of professional nurses in their work environment (Bimenyimana et al. 2009; Yildirim 2009). Yildirim (2009) stated that nurses ran a high risk of being exposed to violence in the work environment. The bullying amongst psychiatric nurses focused more on nurses in their work environment and the negative implications of this form of aggression (Sa & Fleming 2008; Yildirim 2009).

Antoniazzi (2011) stated in a study of respect, as experienced by registered nurses, that violence and aggression were becoming more acceptable in many work environments. Findings revealed that communication was a key factor in conveying respect, including what was and what was not communicated as well as how communication took place amongst colleagues. Yildirim (2009) explained that verbal aggression was the most common type of violence amongst health care personnel; this form of aggression included shouting, reprimanding and belittling. Gossiping had a devastating effect on the work environment (Antoniazzi 2011).

Research reported that education and socialisation at different levels exhibit different values and perceptions of professional identity. This had been observed to create conflict amongst nurses when they enter the workforce (Pearson et al. 2006). In multicultural teams, increased levels of relationship conflict were found. The diversity was related to both process and delegation conflict and affected the communication outcomes (Jager & Raich 2011).

Warnock (2008) stated that forms of unprofessional behaviour might be as subtle as unintended disrespect, judgement of peers, breaches of confidentiality and dishonesty in the disclosure of adverse events. Yildirim (2009) listed the following: isolation and being excluded as an effect of bullying in the work environment. Lewis, Coursol and Wahl (2002) explained that non-verbal aggression manifested in the work environment as glaring, ridicule, isolation, being excluded as well as withholding of support and information, similar to the results of the current study.

Psychiatric nurses experienced a doubtful, suspicious and distrustful team environment. The aggression that psychiatric nurses experienced affected the nurses at various levels. The research findings indicated that the psychiatric nurses themselves were affected by aggression in the work environment. As individuals, their self-worth and self-esteem were affected. They blamed themselves for the aggression they experienced. They also felt incompetent to perform their duties. The psychiatric nurses questioned the colleagues and found that there was no teamwork amongst them. The experience of aggression became passive and the psychiatric nurses reported a doubtful, suspicious and distrustful team environment. Ultimately, the experience of aggression made the psychiatric nurses question their career, losing their passion for their jobs, and left them demotivated and demoralised.

Lewis et al. (2002) stated that most people would like to deal with negative treatment in the work environment in an indirect manner; thus ignoring the behaviour as well as avoiding the perpetrator or not discussing the issue directly. The researcher explained that, as a result of aggression in the work environment, the following feelings manifest: self-blame, shame, self-deprecation, insecurity, inadequacy and a lack of self-confidence (Lewis et al. 2002). Antoniazzi (2011) emphasised that aggression resulted in a lack of motivation, loss of confidence and reduced self-esteem, depression, anger, anxiety, absenteeism and irritability. Lin et al. (2010) added that job stress could be positively correlated with depression. Thus, stress experiences in the work environment could affect the psychiatric nurses’ mental health.

Happell, Martin and Pinikahana (2003) recognised that the work pertaining to nursing was often stressful and that stress had been identified as one of the reasons for nurses failing to function at an optimum level of effectiveness. Stress and work environment conflict had a significant effect on the nurses. As a result, they might experience physiological, psychological and social challenges; thus affecting the individual nurse’s self-esteem and self-confidence that could lead to burnout.

Yildirim (2009) further explained that individuals who were exposed to work environment conflict became unable to do their work because of the damage inflicted on them. Bilgin (2009) explained that conflict in the work environment not only affected service delivery, but also the caring role of nurses. The ward’s culture, insufficient teamwork, a lack of support from administrators and no conflict-prevention training added to the detriment of these nurses. Pearson et al. (2006) added that nurses were unwilling to take responsibility for their own actions. Bullying, violence, damaging effects, harassment and conflicts were some of the words used in various research studies to describe aggression in the work environment. Aggression affected the individuals not just at the physical level, but also at the emotional level. These affects bring into question their job satisfaction, work performance, motivation and productivity (Bilgin 2009; Lewis et al. 2002; Yildirim 2009). Dormann and Zapf (2001) maintained that the indicated job dissatisfaction was closely related to absenteeism, fluctuation, organisational inefficiency and counterproductive behaviour. See the theme aggression on page 2.

The psychiatric nurses experienced limited support when aggression was not acknowledged by colleagues and management. In all the interviews, conducted with the psychiatric nurses, they reported on the evident lack of support in the work environment. They described the lack of support as colleagues or management not acknowledging aggression. The psychiatric nurses experienced that management reprimanded them by constantly reporting the nurses and assigning duties unfairly. Threats of disciplinary action against the psychiatric nurses were a common occurrence. In addition, no recognition would be shown for the nurses’ hard work.

Bilgin (2009) listed occupational and emotional stressors relating to nurses in the work environment and pointed out that little emotional and/or administrative support contributed to the nurses’ stressors. This could negatively affect the nurses by influencing their well-being. Currid (2009) confirmed that a lack of support in the work environment was a source of stress. Nursing staff mostly felt unsupported by managers. Funakoshi, Miyamoto and Kayama (2007) stated that, in order for psychiatric nurses to feel some emotional support, they needed to disclose their concerns openly amongst colleagues. This support was essential and helped the psychiatric nurses not to feel isolated, but as a result feel more supported. Given the theme discussed in 10.3, page 5 (Psychiatric nurses experienced limited support when aggression was not acknowledged by colleagues and management) this reality was clearly absent with detrimental effects. Currid (2009) continued to explain that support could be offered in various forms and stated that clinical supervision was a resource of support in the work environment.

Bowler et al. (2011) explained that punishment or punitive behaviour by management was seen as abusive supervision. This type of behaviour had a negative impact on the nurses.

The nurses would be more likely to outwardly resist managers. It also brought about deviant behaviour. In addition, the nurses experienced emotional distress that resulted in more work-related or family-related conflict (Bowler et al. 2011).

Antoniazzi (2011) found that, in a working environment with dysfunctional nurse-to-nurse relationships, the lack of respect was evident. This was more prominent when the environment had budgetary constraints, increased workloads, increase of personal expectations and constant changes. Antoniazzi (2011) believes that this resulted in increased job stress, decreased job satisfaction and increased sick time to the extent that nurses left the profession. This in itself added to the lack of resources which, in turn, resulted in a high turnover of personnel in the nursing profession.

Psychiatric nurses experienced using coping and defence mechanisms when confronted with emotional stress (McGibbon, Peter & Gallop 2010) and aggression. The psychiatric nurses’ experience of aggression in the above forms resulted in emotional distress, leaving them with feelings of frustration disappointment, mistrust and resentment. As mentioned, experience of emotional distress resulted in the psychiatric nurses using defence mechanisms. Non-mature defence mechanisms differ from mature defence mechanisms. The former involves suppression, neurotic defences such as withdrawal and isolation, narcissistic defences such as projection of anger and immature defences such as passive aggressive behaviour (Kaplan & Sadock 2007). Ultimately the passive manner in which the psychiatric nurses managed aggression was similar to how they experienced aggression amongst colleagues in the work environment.

Burnout, according to Sherring and Knight (2009), occurred when stress was experienced over a prolonged period. Research indicated that stress and burnout were directly related to conflict and aggression in the work environment (Coffey & Coleman 2001). Stress affected the health and well-being of the nurses (Andrews & Wan 2009). According to Hannigan et al. (2000), emotional stressors were described as emotional exhaustion and occurred as a result of feeling emotionally overwhelmed by one’s work. The researchers explained that this might lead to work responsibilities that overwhelm nurses.

Currid (2009) stated that when nurses were overwhelmed by work demands, stressors such as aggression could threaten their ability to cope. Lin et al. (2010) defined coping as constantly changing cognitive and behavioural efforts to manage specific internal and external demands that were appraised as exceeding the resources of the person. Coping was categorised as active coping and avoidant coping in order to manage conflict and stressful events. Isolation took place when the nurses separated themselves from the aggression they experienced in order to cope with it.

Andrews and Wan (2009) explained that when nursing staff experienced stressors in the work environment as unchangeable, it became threatening to them and they used emotional responses such as anger and avoidance as a coping strategy. Lewis et al. (2002) reported that more passive and non-fatal forms of aggression were manifested in the work environment. The researcher explained that passive aggression in the work environment was seen as spreading rumours and not providing information or necessary support. Cottrell (2001) stated that stress and aggression in the work environment compromised factors such as lack of productivity owing to staff conflicts, recruitment and retention challenges, burnout, absenteeism, litigation and rapid staff turnover.

## Limitations

A possible limitation could be that this was a contextual study. The researcher provided a description of the demographics of the participants and a rich description of the results supported by direct quotations of the participants to ensure transferability to a similar context with similar participants.

## Conclusions

In conclusion, the process by which psychiatric nurses experienced aggression in the work environment amongst colleagues was seen as a continuous process. When the psychiatric nurses experienced aggression, it affected them, their ability to perform as a team as well as their daily tasks and duties. When aggression is not acknowledged by not talking about it or addressing the source of aggression, the psychiatric nurses experienced limited support from their colleagues and management who they see as a part of the nursing team. The limited support in the work environment resulted in emotional distress. When the psychiatric nurses experienced emotional distress, they resorted to using defence mechanisms in order to cope with the emotional distress. Different defence mechanisms might be used. If the psychiatric nurses do not have effective support and guidance in the work environment, they would not know how to cope with the distress they experience. At times, destructive coping mechanisms might be used, for example passive aggressive behaviour. This behaviour then once more contributed to the aggression experienced in the work environment. It is clear that attention should be given to create opportunities for psychiatric nurses to master the management of experienced aggression from colleagues.
